# Nongenetic paternal effects via seminal fluid

**DOI:** 10.1002/evl3.124

**Published:** 2019-07-08

**Authors:** Leigh W. Simmons, Maxine Lovegrove

**Affiliations:** ^1^ Centre for Evolutionary Biology School of Biological Sciences The University of Western Australia Crawley 6009 Australia

**Keywords:** Ejaculates, embryo viability, heritability, sperm competition, *Teleogryllus oceanicus*

## Abstract

Mounting evidence suggests that nongenetic paternal effects on offspring may be widespread among animal taxa, but the mechanisms underlying this form of nongenetic inheritance are not yet fully understood. Here, we show that seminal fluids underlie paternal effects on early offspring survival in an insect, the cricket *Teleogryllus oceanicus*, and quantify the contribution of this paternal effect to the inheritance of this important fitness trait. We used castrated males within a full‐sib half‐sib experimental design to show that seminal fluid donors were responsible for variation in the survival of developing embryos to hatching, and in their subsequent survival to adulthood. Increased expression of two seminal fluid protein genes, previously found to be positively associated with sperm quality, was found to be negatively associated with embryo survival. These nongenetic paternal effects hold important implications for the evolution of adaptive maternal responses to sperm competition, and more broadly for the interpretation of sire effects from classic quantitative genetic breeding designs.

**Impact Summary**

The resemblance of offspring to their parents is generally attributed to the contribution of genes packaged in sperm and eggs. However, there is increasing evidence that the environment experienced by parents can also influence the behavior, morphology, and physiology of their offspring. The mechanisms underlying these nongenetic effects are slowly being uncovered. In this study, we asked whether the seminal fluid in ejaculates could affect the survival and performance of offspring. We allowed female crickets to mate with both a normal sperm donating male and a castrated male that transferred only seminal fluid. We found that seminal fluid donors had a strong and significant effect on the survival of offspring but not on adult offspring size or reproductive capacity. The nongenetic paternal effect of seminal fluid donors on offspring survival was sufficient to account for previous estimates of genetic variance in this important fitness trait. Paternal effects have important implications for our understanding of evolutionary processes. Our findings cast doubt on traditional breeding designs aimed at estimating the genetic effects of fathers on offspring, and challenge our views on the nature of inheritance.

Although the adaptive significance of maternal effects have been the subject of considerable research effort (Mousseau and Fox 1998), the potential for paternal effects to influence offspring phenotype was thought to be limited only to species with paternal care (Hunt and Simmons 2000; Stein and Bell 2014). Mounting evidence now points to paternal effects being taxonomically widespread, and to occur through mechanisms other than direct interactions between fathers and their offspring (Curley et al. 2011; Crean and Bonduriansky 2014; Evans et al. 2017). For example, in vertebrates and invertebrates alike, paternal infection can result in transgenerational upregulation of immune function in offspring (McNamara et al. 2014), or in their ability to tolerate parasitic infections (Kaufmann et al. 2014). In rats, exposure of males to endocrine disruptors during their early development can have significant effects on a variety of physiological pathologies, including male fertility, that are observable in their descendants for as many as four generations (Anyway et al. 2005), whereas in humans, paternal obesity, smoking, and alcohol exposure have all been linked to the health of future offspring and grand‐offspring (Soubry et al. 2014). Such transgenerational effects of the paternal environment, or nongenetic inheritance, challenge our views on the very nature of inheritance (Bonduriansky 2012).

There is growing evidence that nongenetic paternal effects on offspring can be mediated by the ejaculate (Evans et al. 2019). The ejaculate consists of both sperm and seminal fluid fractions, both of which can affect offspring phenotype by mechanisms that are independent of the DNA carried in the sperm nucleus. For example, work with house mice has provided evidence that RNAs packaged within sperm can be responsible for transgenerational inheritance of behavioral and metabolic responses to variation in the paternal environment (Gapp et al. 2014; Bohacek and Mansuy 2015; Benito et al. 2018). Studies of rodents have shown that seminal fluid deficiency can affect the growth and health of adult offspring, either directly through the effect of seminal fluid on the integrity of sperm DNA (O et al. 1988) or indirectly through its effects on maternal reproductive tract physiology (Bromfield et al. 2014). Seminal fluid composition can be strongly affected by a male's environment. In molluscs, insects and house mice, a male's social environment generates variation in the protein composition of the seminal fluid; specifically, males will modulate the production of seminal fluid proteins in the ejaculate that contribute to competitive fertilization success when they are exposed to rivals (Ramm et al. 2015; Simmons and Lovegrove 2017; Sloan et al. 2018; Nakadera et al. 2019). Seminal fluid composition can also vary with male age (Simmons et al. 2014) and nutritional status (Binder et al. 2015). Variation in seminal fluid composition may thereby represent an important mechanism by which environmental effects on fathers can be transmitted to their offspring (Crean et al. 2016; Watkins et al. 2018). Indeed, work on insects suggests that seminal fluid may contribute to nongenetic paternal effects on early life survival, adult body condition, and reproductive success (Bonduriansky and Head 2007; García‐González and Simmons 2007; Priest et al. 2008; Crean et al. 2014; Polak et al. 2017; Pascoal et al. 2018).

Nongenetic paternal effects hold significant evolutionary implications. Evolutionary responses to selection depend largely on the levels of genetic variation and the intensity of selection imposed. Quantitative genetic analyses have been widely adopted to estimate the levels of genetic variation in phenotypic traits, by statistically modeling the covariation in phenotypic traits among relatives with the assumption that variation among sires corresponds to additive genetic variation. However, if fathers can contribute to their offspring by mechanisms other than the direct effect of genes, then these paternal effects will inflate estimates of additive genetic variance. Although recent discussions have recognized the necessity to consider nongenetic paternal effects in estimating paternal genetic effects (Banta and Richards 2018), the magnitude of the potential problem has not yet been examined formally (Evans et al. 2019).

We have good evidence to suggest that paternal effects may be an important mechanism of nongenetic inheritance in the cricket *Teleogryllus oceanicus*. Quantitative genetic studies found that the survival of developing embryos to successful hatching exhibited significant among‐sire variance, suggestive of additive genetic variance for early life fitness (García‐González and Simmons 2005a). However, the sire effect on embryo survival was found to be genetically correlated with male investment into reproductive accessory glands, implicating seminal fluid proteins as a potential mechanism mediating paternal effects on this important fitness trait. Indeed, subsequent work confirmed that the paternal effect on embryo survival must have a nongenetic component because when a female mates with two males, a male that imparts high survival to his own offspring also imparts high survival to the offspring sired by his sperm competitor (García‐González and Simmons 2007). Moreover, unlike the case for social insects where seminal fluids are purported to have negative impacts on rival sperm (den Boer et al. 2010), in *T. oceanicus* seminal fluids increase the viability of own and rival sperm (Simmons and Beveridge 2011). Here, using castrated males within a quantitative genetic breeding design, we formally quantified the magnitude of seminal fluid‐derived paternal effects on offspring, and identified two seminal fluid proteins that might contribute to this paternal effect.

Females (dams) in our breeding design received seminal fluid from a castrated outbred male seminal fluid donor (sfd) and a complete ejaculate (sperm and seminal fluid) from a highly inbred isofemale line genetic sire. Any variation in offspring phenotypes due to sfds can be ascribed to nongenetic seminal fluid effects because these donors did not transfer sperm. Any variation due to dams would include maternal and paternal genetic and environmental effects. The maternal and paternal genetic effects were minimized in our breeding design by using dams and sires from a single highly inbred isofemale line.

## Methods

### CASTRATED SEMINAL FLUID DONORS

Seminal fluid donors used in these experiments were the first generation offspring of females collected from a tropical fruit plantation in Carnarvon, Western Australia. Crickets were reared en masse until the final nymphal instar at which time they were isolated into individual containers (7 cm × 7 cm × 5 cm) and supplied with cat chow and water ad libitum. Crickets were checked daily for adult emergence.

On the day of adult emergence, the males were castrated. Crickets were housed at 4°C for 30 minutes and then at –20°C for 2 minutes after which each cricket was placed under a dissecting microscope. With the wings held aside with dissection pins, an incision was made in the dorsal cuticle between the second and third abdominal segments using fine scissors. Each testis was removed with forceps and the wound closed with ethyl cyanoacrylate (Loher and Edson 1973; Larson et al. 2012). Following surgery, crickets were placed back in their individual containers. All crickets were held in a constant temperature room maintained at 26°C on a 12:12 light–dark cycle. Males were used in experiments 14 days after adult emergence.

Thirteen days after surgery, males to be used the following day were mated to a random female from the stock population to ensure they were capable of mating. Once mating had occurred, the spermatophore was collected from the female and its contents evacuated into 20 µL phosphate buffered saline (PBS, Astral Scientific, Taren Point, NSW, Australia) on a microscope slide. A coverslip was placed on to the slide and the contents viewed under a microscope to ascertain whether any sperm were present. The majority of spermatophores were found to be azoospermic. However, on the rare occasion where sperm were detected, these males were discarded. A total of 74 azoospermic males were used as seminal fluid donors.

### ISOFEMALE LINE

To minimize genetic variation among females and fertile males, we created a single isofemale line following a full‐sib mating protocol. One newly emerged adult male and one newly emerged virgin female were taken from the stock culture and housed in a 5‐L container provided with egg carton for substrate, and cat chow and water ad libitum. A moist pad of cotton wool was provided as an oviposition substrate. Eggs were collected after 7 days and nymphs collected as they began to hatch, 14 days after eggs were laid. Full siblings were reared en masse in a 50‐L container, provided with egg carton for substrate, and cat chow and water ad libitum. When crickets started to emerge as adults, a single brother–sister pair was isolated in a 5‐L container and left to mate and lay eggs. The offspring from this pair were reared en masse as per the previous generation. The isofemale line was thus bred via single brother–sister pairing for a total of seven generations, after which the intensity of inbreeding was relaxed. From generation 8 onward, when adult crickets emerged they were left to interact en masse in their 50‐L container and eggs were collected from the group to source subsequent generations.

Crickets for this experiment were sourced from generation 15 of the isofemale line. Male and female crickets were taken at the final nymphal stage. Males were housed in individual containers (7 cm × 7 cm × 5 cm) supplied with cat chow and water ad libitum. Females were housed in groups of 20–30 in 5‐L containers. The day before males were to be used in experiments, they were provided with a virgin female sourced from the stock to ensure they were capable of mating. Crickets were used in experiments 14 days after adult emergence.

### BREEDING DESIGN

Seventy‐four castrated seminal fluid donors (sfd) were each assigned two females from the isofemale line and allowed to mate with both females twice. All matings were observed to ensure the spermatophores remained attached for 60 minutes, the time required for complete transfer of the ejaculate (Simmons et al. 2003). After mating, sfds were frozen at –20°C. Each female was then allocated a brother from the isofemale line and allowed to mate twice, again ensuring that spermatophores remained attached for 60 minutes to allow for complete insemination. Thus, each female received sperm and seminal fluid from a highly inbred full sibling male and seminal fluid from an outbred first generation field‐derived seminal fluid donor.

### EMBRYO SURVIVAL

After mating, females were housed in individual containers provided with a dish of moist sand as an oviposition substrate, and left for 7 days. Eggs were then separated from the sand with water. Fertilization rates exceed 90% and eggs begin to develop within 2 days of laying, evidenced by a 2‐ to 3‐fold increase in volume (Simmons 2001; García‐González and Simmons 2007). Eye spots appear after 7–10 days of development and embryos hatch after 14–19 days. Embryo mortality occurs predominantly between the eye‐spot and hatching stage (Simmons 2001; García‐González and Simmons 2007). By collected eggs after seven days of laying, we were able to sample only eggs that contained developing embryos. Two sets of 50 developing eggs were each placed on damp cotton wool in separate containers. Any remaining eggs were placed en masse in a third container and the eggs incubated at 26°C. Boxes containing 50 developing eggs were checked daily for hatchlings, which were counted and removed each day until no further offspring emerged. Emerged offspring were combined with those that had emerged from the uncounted eggs and these were raised to adulthood. To estimate the embryo survival we would expect from matings between fertile males and females from the isofemale line, we allowed 25 females to mate twice with one brother and quantified embryo survival as described above.

### ADULT OFFSPRING TRAITS

We raised families of offspring to adulthood in 5‐L boxes as described for the parental generation. Males and females were separated at the final instar and monitored daily for adult emergence. On emergence, males were housed in individual containers (7 cm × 7 cm × 5 cm) and females were housed either individually or if there were more than five on any given day, collectively in a 5‐L container with ad libitum food and water.

Fourteen days after adult emergence, males were assayed for sperm viability using the Live/Dead sperm viability kit (Life Technologies, Carlsbad, CA) which stains live sperm green and dead sperm red. Competitive fertilization success is determined largely by sperm viability, which provides an estimate of expected male reproductive performance (García‐González and Simmons 2005b). We removed the spermatophore from a male's genital pouch and ruptured it in 20 µL of Beadle saline (128.3 mM NaCl, 4.7 mM KCl, and 23 mM CaCl_2_) using fine forceps. The sample was mixed thoroughly before taking a 5 µL sample and mixing it with an equal volume of 1:50 diluted 1 mM SYBR‐14 on a clean slide. The sample was left in the dark for 10 minutes before adding 2 µL of 2.4 mM of propidium iodide. Following a further 10‐minute incubation period, sperm were scored under a fluorescence microscope at 20× magnification. Sperm viability was estimated as the number of live sperm divided by the total number of sperm counted that was set at 500. Males were then frozen and later body weight and pronotum width were recorded. When females were 14 days post adult emergence, they were frozen and later dissected to record ovary weight. Ovary weight is a strong predictor of fecundity, and so provides a measure of expected female reproductive performance (Simmons and García‐González 2007). Body weight and pronotum width were recorded prior to dissection.

### SEMINAL FLUID GENE EXPRESSION

Seminal fluid donors were thawed and dissected. Their accessory glands were removed and placed in RNA later (Life Technologies). RNA was then extracted from the entire accessory gland using the PureLink RNA mini kit (Life Technologies) following the manufacturer's instructions. Disruption of the tissue was achieved using a micropestle (Interpath) and column homogenizer (Life Technologies). An on‐column PureLink DNase treatment was used to remove DNA from the sample and the RNA was quantified using a Qubit fluorometer v2.0 (Life Technologies). RNA yield varied from 355 to 1193 ng/µL in a 100 µL elution volume. A total of 2 µg of RNA from each sample was then converted to cDNA using the high‐capacity RNA‐to‐cDNA kit (Life Technologies) following the manufacturer's instructions. The cDNA was diluted to 10 ng/µL using previous data on standard curves to determine input cDNA amount (Simmons and Lovegrove 2017).

Expression assays were conducted for three seminal fluid protein genes; *ToSfp001* (isotig1262), *ToSfp011* (isotig1709), and *ToSfp017* (isotig5129) (Simmons et al. 2013). These genes were chosen because previous studies have found their expression to be associated with ejaculate quality (Simmons et al. 2014; Simmons and Lovegrove 2017). Full methods can be found in Simmons & Lovegrove (2017). In brief, we used previously designed TaqMan custom assay mixes (Life Technologies) and actin as the reference gene. Each assay was run in triplicate for each seminal fluid gene and reference gene with negative controls (no cDNA) on each plate as follows: 1 µL cDNA, 5 µL 2× TaqMan gene expression master mix and 0.5 µL 20× TaqMan custom assay mix in a 10 µL reaction volume. The assays were run on a StepOne Plus Real‐Time PCR machine (Life Technologies) in compatible 96‐well plates using the following cycling conditions: 50°C for 2 minutes and 95°C for 10 minutes, and then 40 cycles of 95°C for 15 seconds and 60°C for 1 minute. Results were analyzed using the StepOne software version 2.3 and then exported into DataAssist version 3.0 software for sample comparison using the comparative C_T_ method (Schmittgen and Livak 2008). Gene expression data were log transformed for statistical analyses.

## Results

**Figure 1:**
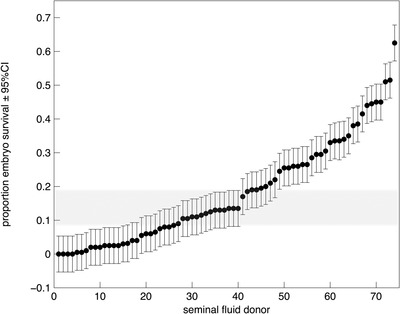
The mean proportion of embryos surviving to hatching across dams mated to each of 74 castrated seminal fluid donors. Seminal fluid donors are ranked by embryo survival and error bars represent the 95% confidence intervals on the proportion. The shaded bar represents the 95% confidence intervals for the proportion of embryos surviving to hatching for 25 isofemale line females each mated to a single isofemale line male.

The mean (±SE) proportion of eggs that hatched varied considerably across dams (range 0–0.63) with a mean (±SE) of 0.18 ± 0.02. We analyzed each individual offspring's egg‐to‐hatch survival in a generalized linear mixed‐effects model using the lme4 package in the R statistical framework (Bates et al. 2015), entering sfd and dam as random effects. Tests of significance were made using likelihood ratio tests. The probability of an embryo surviving to hatching varied significantly among sfds (Table 1, Fig. 1) and there was also significant variation among dams (Table 1). Our breeding design allowed us to use the variance components in Table 1 to calculate the magnitude of the heritability of embryo survival that would have been ascribed to seminal fluid donors had they contributed genetically to their offspring. We calculated this nongenetic paternal effect “heritability” on the liability scale following the procedures outlined for generalized mixed models in de Villemereuil et al. (2016). Standard error was calculated by jacknifing across sfds and used to calculate 95% confidence intervals (CIs) on the estimated nongenetic heritability. The nongenetic paternal effect “heritability” was 0.636 (95% CI, 0.708–0.564).

**Table 1:** Linear mixed models and variance components for the effects of seminal fluid donors (sfd) and dams on embryo survival and the phenotypic traits of adult offspring

					Log likelihood *χ* ^2^ (*P* with df = 1)	
Trait	Offspring sex	Mean ± SE	V_sfd_	V_dam_	sfd	dam
Embryo survival	Combined	0.18 ± 0.02	0.319	0.686	6.83 (<0.001)	1180 (<0.001)
Pronotum width (mm)	Male	6.15 ± 0.01	2.97 × 10^–9^	1.29 × 10^–2^	0.00 (1.000)	31.68 (<0.001)
	Female	5.79 ± 0.01	0.006	0.012	1.33 (0.249)	47.43 (<0.001)
Weight (g)	Male	627.9 ± 3.7	93.19	832.07	0.01 (0.919)	13.76 (<0.001)
	Female	772.8 ± 3.9	0	2744.0	0.00 (1.000)	54.97 (<0.001)
Sperm viability (*P*)	Male	0.68 ± 0.00	2.36 × 10^–10^	6.39 × 10^–4^	0.00 (1.000)	9.81 (0.002)
Ovary weight (g)	Female	188.8 ± 1.9	0	393.8	0.00 (1.000)	44.68 (<0.001)

**Figure 2:**
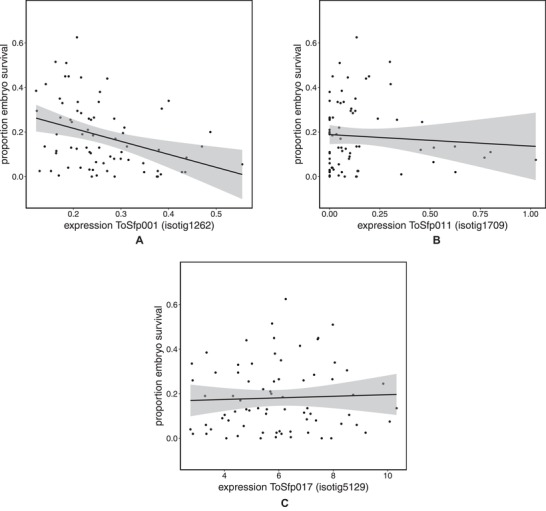
Mean proportion of embryos surviving to hatching for dams mated to each of 74 seminal fluid donors plotted against the expression of three seminal fluid protein genes in the donor's accessory glands.

We next calculated a mean embryo survival for each sfd across the dams with whom he had mated, and used multiple regression analysis to look for associations between embryo survival and seminal fluid gene expression in the accessory glands of sfds (*F*_3,70_ = 5.45, *P* = 0.002; Fig. 2). Donor family mean embryo survival decreased with increasing expression of *ToSfp001* (isotig1262; *F*_1,70_ = 7.71, *P* = 0.007) and to a lesser extent *ToSfp011* (isotig1709; *F*_1,70_ = 4.81, *P* = 0.032) but was not associated with the expression of *ToSfp017* (isotig5129; *F*_1,70_ = 0.86, *P* = 0.355).

A total of 97 of the original 148 dam families provided hatchlings, but of these only 38 dam families survived to adulthood. One to 16 male offspring per dam family and 2–42 female offspring per dam family were available for adult phenotyping. We used general linear mixed models to analyze adult offspring traits, again entering sfd and dam as random effects. However, we found no significant effects of sfds on any aspect of adult offspring performance that we measured, which was largely explained by dam effects (Table 1).

Although our experiment was not designed to examine hatchling‐adult survival, given the high mortality observed among dam families, we modeled the effect of sfd on the probability of obtaining any adult offspring from the females with whom they had mated. There was a significant effect of sfd on the probability that their females produced surviving adult offspring (*χ* ^2^ = 63.54, df 1, *P* < 0.001). We used a generalized linear model with a binomial distribution and probit link function to model the effect of seminal fluid gene expression in the accessory glands of sfds on hatchling‐adult offspring survival. Neither *ToSfp001* (isotig1262) expression (estimate –0.296; 95% CI, –3.35 to 4.24; *χ* ^2^ = 0.02, df 1, *P* = 0.877) nor *ToSfp011* (isotig1709) expression (estimate –0.278; 95% CI, –0.801 to 0.1.376; *χ* ^2^ = 0.03, df 1, *P* = 0.615) influenced the probability that hatched offspring survived to adulthood. We note, however, that the expression of *ToSfp017* (isotig5129) had a nonsignificant negative effect on survival to adulthood with confidence intervals that were highly asymmetric around zero (estimate –0.16; 95% CI, –0.345 to 0.009; *χ* ^2^ = 3.28, df 1, *P* = 0.064).

## Discussion

By mating females to azoospermic males, we have documented nongenetic paternal effects on offspring survival that are mediated by seminal fluid. We found no evidence that seminal fluid affected the body size or reproductive capacity of adult offspring.

It can be difficult to establish whether a parental effect is a maternal effect, a paternal effect, or an interaction between maternal and paternal effects (Crean and Bonduriansky 2014). In species that lack parental care, maternal effects on offspring growth and development are due largely to the amount of resources females allocate to their eggs. Such maternal effects are well documented in insect life histories (Mousseau and Dingle 1991). At least four mechanisms could account for the paternal effects on embryo survival that we have documented here. First, they could in fact be maternal effects. Females of a number of species have been demonstrated to adjust their expenditure on reproduction relative to the perceived quality of their mating partners, so called differential allocation (Sheldon 2000). Thus females could assess males based on, say, the attractiveness of courtship behavior and increase their allocation to eggs after mating with attractive males. Thus, it could be that variation in male attractiveness across seminal fluid donors was responsible for variation in maternal allocation to eggs with causal effects on embryo viability and subsequent offspring survival to adulthood. However, we also showed that variation in the expression of at least two seminal fluid genes in the accessory glands of sfds was associated with embryo viability, and seminal fluid proteins have been found previously to be incorporated into eggs in this species (Simmons 2011). Together, these findings suggest that seminal fluid proteins are involved in this paternal effect. Second, some seminal fluid proteins are known to have gonadotropic effects on females following mating (Avila et al. 2011). Variation in the potency of seminal fluid proteins in stimulating females to allocate more resources to egg production could generate variation in offspring viability among seminal fluid donors. Paternal effects could thus act indirectly through their interaction with maternal effects. Previous studies of *T. oceanicus* have used radioisotope labeled proteins to track both male and female contributions to eggs (Simmons 2011). Although labeled proteins from the ejaculate were found to be incorporated into the female's somatic tissue, ejaculate proteins were incorporated into eggs at 1.6 times the rate of labeled female proteins (ejaculate‐derived proteins 2.5%^−day^, female‐derived proteins 1.5%^−day^) suggesting that seminal fluid‐derived proteins are incorporated directly into developing eggs, rather than affecting maternal allocation (Simmons 2011). Third, seminal fluid proteins may have their effect via their interaction with sperm. In hamsters, ablation of the male accessory glands responsible for the secretion of seminal fluid proteins has the effect of increasing DNA damage to sperm with consequences for rates of implantation and postimplantation mortality of the zygotes they sire (O et al. 1988; Chen et al. 2002; O et al. 2006). Seminal fluid proteins, and particularly ToSfp011 and ToSfp001, are known to influence the viability of sperm in *T. oceanicus* ejaculates (Simmons and Beveridge 2011; Simmons and Lovegrove 2017) and so they might also influence embryo survival through their effects on sperm DNA integrity. Finally, sfps may act directly on developing embryos if they are carried into the egg at the time of fertilization. Interestingly, ToSfp001 binds tightly to sperm, being found in both the sperm and the seminal fluid proteome (Simmons et al. 2013; Simmons et al. 2014), and would be carried into the egg at the time of fertilization where it would have the potential to impact embryo development.

Seminal fluid donors had the capacity to increase and decrease the survival of embryos relative to that expected for the isofemale line males used in this study. This finding replicates that of Garcia‐Gonzalez & Simmons (2007) who found that males who imparted high embryo viability in their own offspring would elevate the viability of their rival's offspring, whereas male's that imparted low viability in their own offspring reduced the viability of their rival's offspring. Collectively these data show that seminal fluids can have both positive and negative effects on embryo development. Nevertheless, Garcia‐Gonzalez and Simmons (2007) found that males capable of elevating embryo viability seemed to have the stronger effect on embryo survival, suggesting that multiple mating by females may serve to capture the positive effects of some males seminal fluids on their offspring, accounting for why polyandry in this species results in a general elevation in embryo survival (Simmons 2001).

We found that the expression of two seminal fluid protein genes in the accessory glands of sfds was negatively associated with the viability of embryos produced by females with whom they mated. Gene expression is indicative of protein production, not its allocation to ejaculates. Nevertheless, previous studies have found that the levels of gene expression are correlated with the amount of protein contained in whole ejaculates (Simmons et al. 2014), and that the expression of *ToSfp011* in the accessory glands is associated with the viability of sperm in whole ejaculates. Thus, male *T. oceanicus* exposed to the calling songs of rivals show an increased expression of *ToSfp011*, of *ToSfp001*, and several other seminal fluid genes that have positive effects on sperm viability (Simmons and Lovegrove 2017). Collectively our findings suggest that increased male expenditure on seminal fluid proteins that affect a male's competitive fertilization success may come at a fitness cost to the offspring he sires. These findings parallel those of two studies, using zebrafish and ascidians, respectively, that have both reported how males reared in high‐risk sperm competition environments sire offspring with reduced survival (Crean et al. 2013; Zajitschek et al. 2014). Moreover, a recent study of zebrafish found that males exposed to rivals had sperm with morphologies that are predicted to promote more efficient fertilization but also had greater levels of DNA damage compared to the sperm of males that were not exposed to rivals, supporting the notion of a trade‐off between male expenditure on gaining fertilizations and on the genetic quality of the offspring they sire (Silva et al. 2019).

Our finding of seminal fluid‐mediated paternal effects holds important implications for quantitative genetic analyses. Half‐sibling breeding designs are widely used for estimating levels of additive genetic variance in behavioral, morphological, and life‐history traits (Falconer and Mackay 1996; Lynch and Walsh 1998). Significant sire effects are assumed to be indicative of additive genetic variance. However, within half‐sibling breeding designs, variance due to sires may in fact arise through nongenetic paternal effects such as those described here. Previously, the heritability of sire induced embryo survival was estimated to be in the region of 0.46 ± 0.29 (García‐González and Simmons 2005a). Our estimate of the magnitude of nongenetic inheritance on the liability scale (0.564–0.708) equates to ∼0.295 on the observed scale, suggesting that much of the heritability in embryo viability estimated previously from a true genetic full‐sib half‐sib design (García‐González and Simmons 2005a) was likely due to nongenetic paternal effects. The strong nongenetic paternal effects on offspring phenotype that we have documented raise general questions around the validity of quantitative genetic designs, such as the full‐sib half‐sib design, that ascribe sire effects to additive genetic variance (Banta and Richards 2018; Evans et al. 2019). They also hold important implications for our understanding of evolutionary processes.

Genetic models for the evolution and maintenance of female mate choice rely on indirect genetic benefits accruing to offspring. However, genetic variation in fitness traits should be eroded by female choice leading to the paradox of the lek (Kirkpatrick and Ryan 1991). An apparent contradiction of the lek paradox is the finding that secondary sexual traits have greater levels of additive genetic variance than do naturally selected traits (Pomiankowski and Møller 1995). Our findings highlight the real possibility that previous estimates of genetic variance in secondary sexual traits may have been greatly inflated by nongenetic paternal effects. This is especially true as the hallmark of secondary sexual traits is their dependence on a male's ability to acquire resources from its environment and to allocate those resources to trait development (Rowe and Houle 1996). As such, secondary sexual trait expression offers a signal of a male's developmental environment and the potential nongenetic effects of that environment on his future offspring. Thus, Bonduriansky and Day (2013) modeled the evolution of costly female preferences in the context of paternal effects, finding that preferences are more likely to be maintained when the benefits of choice are transmitted to offspring via paternal effects that are renewed each generation through environmental effects on males. There is good evidence that ejaculate composition can be condition dependent (Simmons and Kotiaho 2002; Perry and Rowe 2010; Wigby et al. 2016; Patlar et al. 2019), offering an avenue for female choice for paternal effects that promote offspring fitness (Crean et al. 2016; Macartney et al. 2018).

In conclusion, we show that seminal fluids are responsible for a nongenetic paternal effect on the survival of offspring. Nongenetic inheritance holds important implications for our interpretation of quantitative genetic designs that seek to establish the levels of additive genetic variation in phenotypic traits. Nongenetic paternal effects can generate novel and complex evolutionary dynamics. The negative effects on offspring survival of seminal fluid proteins that contribute to a male's competitive fertilization success may hold important implications for female fitness, and the evolution of adaptive maternal responses in the face of sperm competition.

Associate Editor: R. Snook

## References

[evl3124-bib-0001] Anyway, M. D. , A. S. Cupp , M. Uzumcu , and M. K. Skinner . 2005 Epigenetic transgenerational actions of endocrine disruptors and male fertility. Science 308:1466–1469. 15933200 10.1126/science.1108190 PMC11423801

[evl3124-bib-0002] Avila, F. W. , L. K. Sirot , B. A. LaFlamme , C. D. Rubinstein , and M. F. Wolfner . 2011 Insect seminal fluid proteins: identification and function. Ann. Rev. Entomol. 56:21–40. 20868282 10.1146/annurev-ento-120709-144823 PMC3925971

[evl3124-bib-0003] Banta, J. A. , and C. L. Richards . 2018 Quantitative epigenetics and evolution. Heredity 121:210–224. 29980793 10.1038/s41437-018-0114-x PMC6082842

[evl3124-bib-0004] Bates, D. , M. Mächler , B. Bolker , and S. Walker . 2015 Fitting linear mixed‐effects models using lme4. J. Stat. Soft. 67:1–48.

[evl3124-bib-0005] Benito, E. , C. Kerimoglu , B. Ramachandran , T. Pena‐Centeno , G. Jain , R. M. Stilling , et al. 2018 RNA‐dependent intergenerational inheritance of enhanced synaptic plasticity after environmental enrichment. Cell Rep. 23:546–554. 29642011 10.1016/j.celrep.2018.03.059 PMC5912949

[evl3124-bib-0006] Binder, N. K. , J. R. Sheedy , N. J. Hannan , and D. K. Gardner . 2015 Male obesity is associated with changed spermatozoa Cox4i1 mRNA level and altered seminal vesicle fluid composition in a mouse model. Mol. Hum. Repord. 21:424–434. 10.1093/molehr/gav010 25731709

[evl3124-bib-0007] Bohacek, J. , and I. M. Mansuy . 2015 Molecular insights into transgenerational non‐genetic inheritance of acquired behaviours. Nat. Rev. Gen. 16:641–652. 10.1038/nrg3964 26416311

[evl3124-bib-0008] Bonduriansky, R. 2012 Rethinking heredity, again. Trends Ecol. Evol. 27:330–336. 22445060 10.1016/j.tree.2012.02.003

[evl3124-bib-0009] Bonduriansky, R. , and T. Day . 2013 Nongenetic inheritance and the evolution of costly female preference. J. Evol. Biol. 26:76–87. 23163399 10.1111/jeb.12028

[evl3124-bib-0010] Bonduriansky, R. , and M. Head . 2007 Maternal and paternal condition effects on offspring phenotype in *Telostylinus angusticollis* (Diptera: Neriidae). J. Evol. Biol. 20:2379–2388. 17956399 10.1111/j.1420-9101.2007.01419.x

[evl3124-bib-0011] Bromfield, J. J. , J. E. Schjenken , P. Y. Chin , A. S. Care , M. J. Jasper , and S. A. Robertson . 2014 Maternal tract factors contribute to paternal seminal fluid impact on metabolic phenotype in offspring. Proc. Natl. Acad. Sci. USA 111:2200–2205. 24469827 10.1073/pnas.1305609111 PMC3926084

[evl3124-bib-0012] Chen, H. , M. P. L. Cheung , P. H. Chow , A. L. M. Cheung , W. Liu , and W. S. O . 2002 Protection of sperm DNA against oxidative stress in vivo by accessory sex gland secretions in male hamsters. Reproduction 124:491–499. 12361467 10.1530/rep.0.1240491

[evl3124-bib-0013] Crean, A. J. , and R. Bonduriansky . 2014 What is a paternal effect? Trends Ecol. Evol. 29:554–559. 25130305 10.1016/j.tree.2014.07.009

[evl3124-bib-0014] Crean, A. J. , J. M. Dwyer , and D. J. Marshall . 2013 Adaptive paternal effects? Experimental evidence that the paternal environment affects offspring performance. Ecology 94:2575–2582. 24400509 10.1890/13-0184.1

[evl3124-bib-0015] Crean, A. J. , A. M. Kopps , and R. Bonduriansky . 2014 Revisiting telegony: offspring inherit an acquired characteristic of their mother's previous mate. Ecol. Let. 17:1545–1552. 25270393 10.1111/ele.12373 PMC4282758

[evl3124-bib-0016] Crean, A. J. , M. I. Adler , and R. Bonduriansky . 2016 Seminal fluid and mate choice: new predictions. Trends Ecol. Evol. 31:253–255. 26948861 10.1016/j.tree.2016.02.004

[evl3124-bib-0017] Curley, J. P. , R. Mashoodh , and F. A. Champagne . 2011 Epigenetics and the origins of paternal effects. Horm. Behav. 59:306–314. 20620140 10.1016/j.yhbeh.2010.06.018 PMC2975825

[evl3124-bib-0018] de Villemereuil, P. , H. Schielzeth , S. Nakagawa , and M. Morrissey . 2016 General methods for evolutionary quantitative genetic inference from generalized mixed models. Genetics 204:1281–1294. 27591750 10.1534/genetics.115.186536 PMC5105857

[evl3124-bib-0019] den Boer, S. P. A. , B. Baer , and J. J. Boomsma . 2010 Seminal fluid mediates ejaculate competition in social insects. Science 327:1506–1509. 20299595 10.1126/science.1184709

[evl3124-bib-0020] Evans, J. P. , R. A. Lymbery , K. S. Wiid , M. M. Rahman , and C. Gasparini . 2017 Sperm as moderators of environmentally induced paternal effects in a livebearing fish. Biol. Let. 13:20170087. 28404822 10.1098/rsbl.2017.0087 PMC5414698

[evl3124-bib-0021] Evans, J. P. , A. J. Wilson , A. Pilastro , and F. Garcia‐Gonzalez . 2019 Ejaculate‐mediated paternal effects: evidence, mechanisms and evolutionary implications. Reproduction 157:R109–R126. 30668523 10.1530/REP-18-0524

[evl3124-bib-0022] Falconer, D. S. , and T. F. C. Mackay . 1996 Introduction to quantitative genetics. Longman, Harlow, U.K.

[evl3124-bib-0023] Gapp, K. , A. Jawaid , P. Sarkies , J. Bohacek , P. Pelczar , J. Prados , et al. 2014 Implication of sperm RNAs in transgenerational inheritance of the effects of early trauma in mice. Nat. Neurosci. 17:667–669. 24728267 10.1038/nn.3695 PMC4333222

[evl3124-bib-0024] García‐González, F. , and L. W. Simmons . 2005a The evolution of polyandry: intrinsic sire effects contribute to embryo viability. J. Evol. Biol. 18:1097–1103. 16033583 10.1111/j.1420-9101.2005.00889.x

[evl3124-bib-0025] García‐González, F. , and L. W. Simmons 2005b Sperm viability matters in insect sperm competition. Curr. Biol. 15:271–275. 15694313 10.1016/j.cub.2005.01.032

[evl3124-bib-0026] García‐González, F. , and L. W. Simmons 2007 Paternal indirect genetic effects on offspring viability and the benefits of polyandry. Curr. Biol. 17:32–36. 17208183 10.1016/j.cub.2006.10.054

[evl3124-bib-0027] Hunt, J. , and L. W. Simmons . 2000 Maternal and paternal effects on offspring phenotype in the dung beetle *Onthophagus taurus* . Evolution 54:936–941. 10937266 10.1111/j.0014-3820.2000.tb00093.x

[evl3124-bib-0028] Kaufmann, J. , T. L. Lenz , M. Milinski , and C. Eizaguirre . 2014 Experimental parasite infection reveals costs and benefits of paternal effects. Ecol. Let. 17:1409–1417. 25168056 10.1111/ele.12344 PMC4282062

[evl3124-bib-0029] Kirkpatrick, M. , and M. J. Ryan . 1991 The evolution of mating preferences and the paradox of the lek. Nature 350:33–38.

[evl3124-bib-0030] Larson, E. L. , J. A. Andrés , and R. G. Harrison . 2012 Influence of the male ejaculate on post‐mating prezygotic barriers in field crickets. PLoS One 7:e46202. 23071547 10.1371/journal.pone.0046202 PMC3468576

[evl3124-bib-0031] Loher, W. , and K. Edson . 1973 The effect of mating on egg production and release in the cricket *Teleogryllus commodus* . Entomol. Exp. Appl. 16:483–490.

[evl3124-bib-0032] Lynch, M. , and B. Walsh . 1998 Genetics and analysis of quantitative traits. Sinauer, Sunderland, MA.

[evl3124-bib-0033] Macartney, E. L. , A. J. Crean , and R. Bonduriansky . 2018 Epigenetic paternal effects as costly, condition‐dependent traits. Heredity 121:248–256. 29904169 10.1038/s41437-018-0096-8 PMC6082865

[evl3124-bib-0034] McNamara, K. B. , E. van Lieshout , and L. W. Simmons . 2014 The effect of maternal and paternal immune challenge on offspring immunity and reproduction in a cricket. J. Evol. Biol. 27:1020–1028. 24750259 10.1111/jeb.12376

[evl3124-bib-0035] Mousseau, T. A. , and H. Dingle . 1991 Maternal effects in insect life histories. Annu. Rev. Entomol. 36:511–534.

[evl3124-bib-0036] Mousseau, T. A. , and C. W. Fox . 1998 The adaptive significance of maternal effects. Trend Ecol. Evol. 13:403–407. 10.1016/s0169-5347(98)01472-4 21238360

[evl3124-bib-0037] Nakadera, Y. , A. Giannakara , and S. A. Ramm . 2019 Plastic expression of seminal fluid protein genes in a simultaneously hermaphroditic snail. Behav. Ecol. 10.1093/beheco/arz027.

[evl3124-bib-0038] O, W. S. , H. Chen , and P. H. Chow . 2006 Male genital tract antioxidant enzymes: their ability to preserve sperm DNA integrity. Mol. Cell Endocrin. 250:80–83. 10.1016/j.mce.2005.12.029 16442705

[evl3124-bib-0039] O, W. S. , H. Q. Chen , and P. H. Chow . 1988 Effects of male accessory sex gland secretions on early embryonic development in the golden hamster. J. Reprod. Fert. 84:341–344. 10.1530/jrf.0.0840341 3184052

[evl3124-bib-0040] Pascoal, S. , J. M. Jarrett Benjamin , E. Evans , and M. Kilner Rebecca . 2018 Superior stimulation of female fecundity by subordinate males provides a mechanism for telegony. Evol. Let. 2:114–125. 30283669 10.1002/evl3.45 PMC6121788

[evl3124-bib-0041] Patlar, B. , M. Weber , and S. A. Ramm . 2019 Genetic and environmental variation in transcriptional expression of seminal fluid proteins. Heredity 122:595–611. 30356222 10.1038/s41437-018-0160-4 PMC6461930

[evl3124-bib-0042] Perry, J. C. , and L. Rowe . 2010 Condition‐dependent ejaculate size and composition in a ladybird beetle. Proc. R. Soc. Lond. B Biol. Sci. 277:3639–3648. 10.1098/rspb.2010.0810 PMC2982242 20573622

[evl3124-bib-0043] Polak, M. , L. W. Simmons , J. B. Benoit , K. Ruohonen , S. J. Simpson , and S. M. Solon‐Biet . 2017 Nutritional geometry of paternal effects on embryo mortality. Proc. R. Soc. Lond. B Biol. Sci. 284:20171492. 10.1098/rspb.2017.1492 PMC5647300 29021174

[evl3124-bib-0044] Pomiankowski, A. , and A. P. Møller . 1995 A resolution of the lek paradox. Proc. R. Soc. Lond. B Biol. Sci. 260:21–29.

[evl3124-bib-0045] Priest, N. K. , D. A. Roach , and L. F. Galloway . 2008 Cross‐generational fitness benefits of mating and male seminal fluid. Biol. Let. 4:6–8. 17986427 10.1098/rsbl.2007.0473 PMC2412926

[evl3124-bib-0046] Ramm, S. , D. Edward , A. Claydon , D. Hammond , P. Brownridge , J. Hurst , et al. 2015 Sperm competition risk drives plasticity in seminal fluid composition. BMC Biol. 13:87. 26507392 10.1186/s12915-015-0197-2 PMC4624372

[evl3124-bib-0047] Rowe, L. , and D. Houle . 1996 The lek paradox and the capture of genetic variance by condition dependent traits. Proc. R. Soc. Lond. B Biol. Sci. 263:1415–1421.

[evl3124-bib-0048] Schmittgen, T. D. , and K. J. Livak . 2008 Analysing real‐time PCR data by the comparative CT method. Nat. Prot. 3:1101–1108. 10.1038/nprot.2008.73 18546601

[evl3124-bib-0049] Sheldon, B. C. 2000 Differential allocation: tests, mechanisms and implications. Trend Ecol. Evol. 15:397–402. 10.1016/s0169-5347(00)01953-4 10998516

[evl3124-bib-0050] Silva, W. T. A. F. , P. Sáez‐Espinosa , S. Torijo‐Boix , A. Romero , C. Devaux , M. Durieux , et al. 2019 The effects of male social environment on sperm phenotype and genome integrity. J. Evol. Biol. 32:535–544. 30817032 10.1111/jeb.13435 PMC6850410

[evl3124-bib-0051] Simmons, L. W. 2001 The evolution of polyandry: an examination of the genetic incompatibility and good‐sperm hypotheses. J. Evol. Biol. 14:585–594.

[evl3124-bib-0052] Simmons, L. W. 2011 Allocation of maternal‐ and ejaculate‐derived proteins to reproduction in female crickets, *Teleogryllus oceanicus* . J. Evol. Biol. 24:132–138. 21044201 10.1111/j.1420-9101.2010.02158.x

[evl3124-bib-0053] Simmons, L. W. , and M. Beveridge . 2011 Seminal fluid affects sperm viability in a cricket. PLoS One 6:e17975. 21455309 10.1371/journal.pone.0017975 PMC3063794

[evl3124-bib-0054] Simmons, L. W. , and F. García‐González . 2007 Female crickets trade offspring viability for fecundity. J. Evol. Biol. 20:1617–1623. 17584254 10.1111/j.1420-9101.2007.01320.x

[evl3124-bib-0055] Simmons, L. W. , and J. S. Kotiaho . 2002 Evolution of ejaculates: patterns of phenotypic and genotypic variation and condition dependence in sperm competition traits. Evolution. 56:1622–1631. 12353755 10.1111/j.0014-3820.2002.tb01474.x

[evl3124-bib-0056] Simmons, L. W. , and M. Lovegrove . 2017 Socially‐cued seminal fluid gene expression mediates responses in ejaculate quality to sperm competition risk. Proc. R. Soc. Lond. B Biol. Sci. 284:20171486. 10.1098/rspb.2017.1486 PMC5577498 28855372

[evl3124-bib-0057] Simmons, L. W. , J. Wernham , F. García‐González , and D. Kamien . 2003 Variation in paternity in the field cricket *Teleogryllus oceanicus*: no detectable influence of sperm numbers or sperm length. Behav. Ecol. 14:539–545.

[evl3124-bib-0058] Simmons, L. W. , Y.‐F. Tan , and A. H. Millar . 2013 Sperm and seminal fluid proteomes of the field cricket *Teleogryllus oceanicus*: identification of novel proteins transferred to females at mating. Insect Mol. Biol. 22:115–130. 23211034 10.1111/imb.12007

[evl3124-bib-0059] Simmons, L. W. , M. Beveridge , L. Li , Y.‐F. Tan , and A. H. Millar . 2014 Ontogenetic changes in seminal fluid gene expression and the protein composition of cricket seminal fluid. Evol. Dev. 16:101–109. 24617989 10.1111/ede.12068

[evl3124-bib-0060] Sloan, N. S. , M. Lovegrove , and L. W. Simmons . 2018 Social manipulation of sperm competition intensity reduces seminal fluid gene expression. Biol. Let. 14:20170659. 29367215 10.1098/rsbl.2017.0659 PMC5803594

[evl3124-bib-0061] Soubry, A. , C. Hoyo , R. L. Jirtle , and S. K. Murphy . 2014 A paternal environmental legacy: evidence for epigenetic inheritance through the male germ line. Bioessays 36:359–371. 24431278 10.1002/bies.201300113 PMC4047566

[evl3124-bib-0062] Stein, L. R. , and A. M. Bell . 2014 Paternal programming in the sticklebacks. Anim. Behav. 95:165–171. 27011391 10.1016/j.anbehav.2014.07.010 PMC4801484

[evl3124-bib-0063] Watkins, A. J. , I. Dias , H. Tsuro , D. Allen , R. D. Emes , J. Moreton , et al. 2018 Paternal diet programs offspring health through sperm‐ and seminal plasma‐specific pathways in mice. Proc. Natnl. Acad. Sci. USA 115:10064. 10.1073/pnas.1806333115 PMC6176621 30150380

[evl3124-bib-0064] Wigby, S. , J. C. Perry , Y.‐H. Kim , and L. K. Sirot . 2016 Developmental environment mediates male seminal protein investment in *Drosophila melanogaster* . Funct. Ecol. 30:410–419. 27546947 10.1111/1365-2435.12515 PMC4974917

[evl3124-bib-0065] Zajitschek, S. , C. Hotzy , F. Zajitschek , and S. Immler . 2014 Short‐term variation in sperm competition causes sperm‐mediated epigenetic effects on early offspring performance in the zebrafish. Proc. R. Soc. Lond. B Biol. Sci. 281:20140422. 10.1098/rspb.2014.0422 PMC4024299 24789902

